# Challenges of the Biopharmaceutical Industry in the Application of Prescriptive Maintenance in the Industry 4.0 Context: A Comprehensive Literature Review

**DOI:** 10.3390/s24227163

**Published:** 2024-11-07

**Authors:** Johnderson Nogueira de Carvalho, Felipe Rodrigues da Silva, Erick Giovani Sperandio Nascimento

**Affiliations:** 1 Oswaldo Cruz Foundation FIOCRUZ, Rio de Janeiro 21040-900, Brazil; johnderson.carvalho@ba.estudante.senai.br (J.N.d.C.); frodrigues@bio.fiocruz.br (F.R.d.S.); 2 Stricto Sensu Department, SENAI CIMATEC University Center, Salvador 41650-010, Brazil; 3 Surrey Institute for People-Centred Artificial Intelligence, Faculty of Engineering and Physical Sciences, University of Surrey, Guildford GU2 7XH, UK

**Keywords:** prescriptive maintenance, predictive maintenance, machine learning, deep learning, biopharmaceutical industry, pharmaceutical industry, Industry 4.0

## Abstract

The biopharmaceutical industry has specificities related to the optimization of its processes, the effectiveness of the maintenance of the productive park in the face of regulatory requirements. and current concepts of modern industry. Current research on the subject points to investments in the health area using the current tools and concepts of Industry 4.0 (I4.0) with the objective of a more assertive production, reduction of maintenance costs, reduction of operating risks, and minimization of equipment idle time. In this context, this study aims to characterize the current knowledge about the challenges of the biopharmaceutical industry in the application of prescriptive maintenance, which derives from predictive maintenance, in the context of I4.0. To achieve this, a systematic review of the literature was carried out in the scientific knowledge bases IEEE Xplore, Scopus, Web of Science, Science Direct, and Google Scholar, considering works such as Reviews, Article Research, and Conference Abstracts published between 2018 and 2023. The results obtained revealed that prescriptive maintenance offers opportunities for improvement in the production process, such as cost reduction and greater proximity to all actors in the areas of production, maintenance, quality, and management. The limitations presented in the literature include a reduced number of models, the lack of a clearer understanding of its construction, lack of applications directly linked to the biopharmaceutical industry, and lack of measurement of costs and implementation time of these models. There are significant advances in this area including the implementation of more elaborate algorithms used in artificial intelligence neural networks, the advancement of the use of decision support systems as well as the collection of data in a more structured and intelligent way. It is concluded that for the adoption of prescriptive maintenance in the pharmaceutical industry, issues such as the definition of data entry and analysis methods, interoperability between “shop floor” and corporate systems, as well as the integration of technologies existing in the world, must be considered for I4.0.

## 1. Introduction

The production of biomedicines [1] began to gain ground in relation to pharmaceutical chemicals at the end of the Second World War, when knowledge of biotechnology began to play a significant role in the production of medicines and vaccines, and was increasingly becoming an economically strategic area within large pharmaceutical companies. The biopharmaceutical sector produces biological medicines or biopharmaceuticals that generally consist of active substances produced from microorganisms, tissues of animal or plant origin, or genetically modified cell cultures. Some examples of products originating from this industry are antibiotics, vaccines, hormones, biopharmaceuticals, monoclonal antibodies, and reagents, among others.

**Figure 1:**
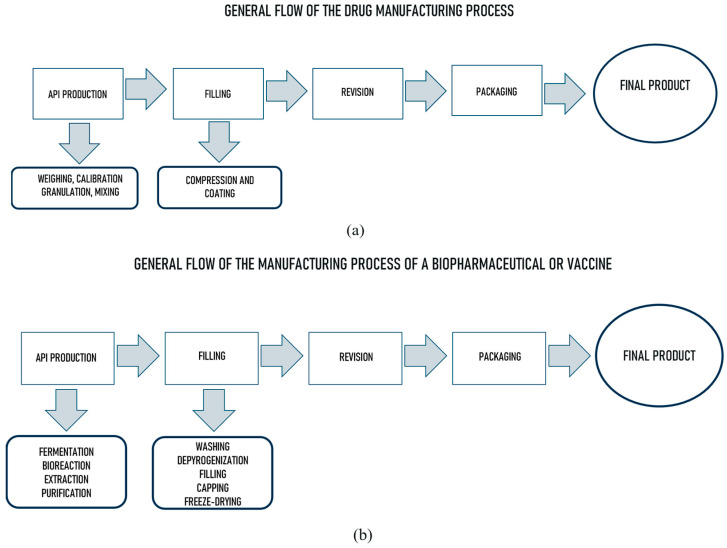
General process flows to produce a drug (**a**) and a biopharmaceutical or vaccine product (**b**).

The production processes [2] carried out in this type of industry have peculiarities that require special care in the implementation of their phases, consisting of stages such as host cell culture, fermentation, extraction, purification, isolation, sterilization, production of active pharmaceutical ingredients, bottling and packaging of the product. Given the high complexity of the processes, this industry has a special need for sophisticated equipment, appropriate utilities, use of clean rooms, strict quality controls, and specific and demanding legislation because of the purpose of the products. Compliance with all safety standards is essential for the product, workers, and the end patient. For equipment that meets the specificities of each phase of the process, there are bioreactors, fermenters, industrial chromatographs, bottling lines, freeze dryers, hydrogen peroxide generators, among many others that rely on rigorous materials in their manufacture and high on-board automation. Regarding utilities, there is a need to use water with very high purification rates (PW and WFI), dry compressed air, ozone, and pure steam, among others. In clean rooms, there is a need to maintain adequate control of temperature, pressure, humidity and the number of particulates in the air. Figure 1 illustrates in general terms the main differences between the production processes of a drug by chemical synthesis and a biopharmaceutical or vaccine by biotechnology.

It is noted that the manufacturing process flows are similar, with differences in the equipment used such as greater complexity, greater monitoring of the biopharmaceutical process, and different regulatory requirements. Therefore, the manufacture of biomedicines requires an extraordinary technological challenge, involving complex activities that include sophisticated bioreaction processes, high-performance purification systems, quality control with highly sensitive methodologies, among other concerns such as avoiding cross-contamination of processes and properly disposing of residues from API production elements in the environment. All these factors, together with the regulatory requirements for validation of computerized systems, make it essential that the biopharmaceutical sector can make use of all the concepts, tools, and solutions available to modern industry.

In [3], an interesting field study was carried out, based on a literature review, on the applications and impacts of Industry 4.0 (I4.0) in the biopharmaceutical sector. This fieldwork, carried out between August 2019 and February 2020, involved semi-structured interviews with managers of pharmaceutical companies and experts in the topic of I4.0. The main results found that there are still cultural barriers to be overcome in the companies themselves in the sector to implement the technologies and solutions available on the market, and a lack of technical knowledge was identified regarding exploring and using the tools available in modern industry to their full capacity. Regulatory requirements for the sector are still considered a challenge when implementing the elements, tools, and solutions proposed by Industry 4.0.

In [4], there is a more in-depth approach to aspects of predictive maintenance (PdM) and applications in artificial intelligence (AI) aimed at the area of supercomputing. The article, in its literature review, noted that there are few studies from 2010 to 2019 in deep learning (DL). A study of the methodologies used and their results is carried out. The greatest contributions to this topic were found between 2017 and 2019. The work identifies the growing use of convolutional and recurrent neural networks (CNNs and RNNs) in the context of predicting and classifying asset failures in a system. Machine learning (ML) techniques are also noted to be used in PdM.

In [5], we see the application of the concepts of PdM and prescriptive maintenance (PsM) in a metallurgical factory, presenting a model without detailing and delving into the tools used and results obtained.

Finally, there is a need to invest in I4.0 applications for the production process of the biopharmaceutical industry sector, due to its importance, relevance, and participation in society. In this context, the objective of this work is to identify and characterize current knowledge on the topic and map challenges of the biopharmaceutical industry in the application of prescriptive maintenance in the context of I4.0. This article is organized as follows: in addition to this Introduction, Section 2 presents important concepts for the study of the topic, Section 3 describes the methodology adopted in the investigation, Section 4 presents and analyzes the results observed and finally, Section 5 includes conclusions and perspectives (or opportunities) for future research are presented.

## 2. Important Concepts

According to Creswell [6], when proposing an investigation, it is important that the researcher identifies and defines the terms that are important for understanding it. Therefore, the following subsections define important terms for this research.

### 2.1. Industry 4.0 and 5.0

**Figure 2:**
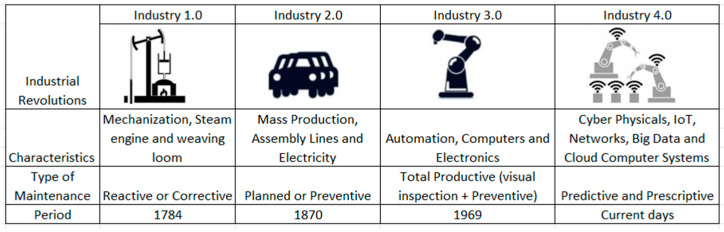
Adapted from [1]. Summary table of industrial revolutions.

Historically, the evolution of the production process over time is contextualized through Figure 2.

According to Schwab [7], Industry 4.0 (I4.0), also called the Fourth Industrial Revolution, began to take shape in 2011, at the Hannover Fair in Germany. It is an expression that encompasses technologies for automation and data exchange, and uses concepts from cyber-physical systems, Internet of Things (IoT), and cloud computing. The focus of the Fourth Industrial Revolution is to improve the efficiency and productivity of processes. For Schwab, the fourth industrial revolution is not limited to intelligent and connected systems and machines. Its scope is much broader, reaching all areas of knowledge, bringing together new technologies and the interaction between physical, digital, and biological domains. This transformation, also known as the Fourth Industrial Revolution, uses some tools and methodologies such as machine learning (ML), IoT, big data, prescriptive maintenance (PsM), and digitalization, which will be the subject of our research.

According to articles [8,9,10,11], the Fifth Industrial Revolution or Industry 5.0 (I5.0) is already beginning, and it conceptually uses the same technologies as I4.0 (IoT, big data, collaborative robots (Cobots), wireless broadband connectivity, etc.) with some updates, aiming to provide more security to data networks, generate a more global and decentralized governance of emerging technologies and the democratization of knowledge co-production from big data, based on the new concept of symmetric innovation. I5.0 is about building complex, hyper-connected digital networks without compromising the long-term security and sustainability of an innovative ecosystem and its constituents. In summary, these articles define the Fifth Industrial Revolution as the one that will do what I4.0 failed to do: promote a fairer and more sustainable society, in which there is a symbiotic/collaborative relationship between the human being and the machine/robot. Concluding this concept, according to [12], we can verify the potential of I5.0 (digital drugs, smart factory, green ecology, compliance with the WHO (World Health Organization) sustainable development goals (SDGs), etc.) and the changes that this industry will bring to society (scalability, human-robot coworking, data security, skilled workforce, etc.).

### 2.2. Prescriptive Maintenance (PsM)

According to [13], we can classify the types of maintenance that exist today into three types:

(a) Corrective maintenance (CM)—is maintenance carried out after the occurrence of a breakdown, designed to put an item in a position to perform a required function. It is known as “putting out fires”. It is a type of maintenance that does not require any prior planning, limiting itself to correcting a failure of a production asset, not measuring the loss of that product being processed nor the final cost used in this type of maintenance.
(b) Preventive maintenance (PM)—all maintenance services carried out on machines that are not failing can be classified as preventive. It is scheduled maintenance and is more efficient than corrective maintenance. This type of maintenance requires planning in the acquisition and identification phases for critical parts to be replaced in production assets, as well as aligning the scheduled stoppage of the asset with production to carry out this type of work.
(c) Predictive maintenance (PdM)—these are preventive maintenance tasks that aim to monitor the machine or parts, through monitoring, measurements or statistical control and try to predict how close the failure will occur. In this type of maintenance, sensors are used in the main components which are considered critical for the production asset to measure and monitor and find possible anomalies and defects during its operation. Examples of techniques used in predictive maintenance are vibration and noise analysis of rotating machines, temperature monitoring of electrical circuits, analysis of the conditions of lubricants used in equipment, measurements of currents, and electrical discharges, among others.

Prescriptive maintenance (PsM) [14] is known as the future of maintenance. It evolved from predictive. Predictive maintenance (PdM) offers constant monitoring with the ability to predict the current and future behavior of assets and, sometimes, some limited recommendations, which are relevant to making improvements and avoiding unscheduled downtime. But in most cases, monitoring only covers devices and does not have the ability to analyze other process variables. Prescriptive maintenance uses forms of AI that can be machine learning or deep learning to gain a broader perspective of the process, making advanced suggestions to improve production, support workflow and reduce downtime. The basic difference is that predictive maintenance predicts when it will fail and what the possible failure is, while the prescriptive one points out how to avoid the failure and reduce the risk of it occurring. In some technical articles it is called the future of maintenance 4.0.

### 2.3. Machine Learning (ML)

According to [15], with the expansion of the use of AI, the growth in the large volume of data generated from various areas of knowledge and the increase in the capacity to process all this information, new tools based on machine learning have emerged. This is a sub-area of artificial intelligence that is part of several of the technologies currently used. It is a process in which computers can learn according to expected responses through associations of different data, which can be images, numbers, digitized information, and everything that this technology can identify.

### 2.4. Deep Learning (DL)

Deep learning is also AI technology, just like machine learning, based on the principle of neural networks, seeking to imitate the human brain with greater precision, like our neurons that receive a large amount of information. The main difference between deep learning and machine learning is that the latter works linearly and the first works in hierarchically linked layers, enabling more complex and in-depth analyses. Among the architectures or types of neural networks used, we include deep neural networks, recurrent neural networks (RNN), long short-term memory networks (LSTM), and convolutional neural networks (CNN), among others.

## 3. Methodology

To identify and characterize current knowledge on the topic and the challenges of the biopharmaceutical industry in the application of PsM in the context of I4.0, a systematic review of the literature was carried out, divided into four stages: Planning, Scope, Evaluation, and Synthesis. The methodological procedure adopted in each of these steps is described in the next subsections.

### 3.1. Planning

In the first stage, the scientific knowledge bases investigated were defined, namely: IEEE Xplore, Scopus, Web of Science, Science Direct and Google Scholar.

The objective of this review is to identify works that offer solutions developed with artificial intelligence algorithms to predict failures in equipment in the biopharmaceutical industry, and that use machine learning techniques to identify research methodologies used in equivalent contexts and their results, in addition to mapping the essential requirements to define the best machine learning technique for applying PsM or PdM in production.

In these databases, works such as Reviews, Article Research, and Conference Abstracts published in the period from 2018 to 2024, until 9 April, were searched, which contained the descriptors and search strings below in the Title, Summary, and Keywords fields:

(“Prescriptive Maintenance” OR “Predictive Maintenance”) AND

(“Machine Learning” OR “Deep Learning”) AND

(“Biopharmaceutical Industry” OR “Pharmaceutical Industry” OR “Industry 4.0”)

### 3.2. Scope

In the next stage, the three guiding questions to be answered to achieve the objective of this study were established:

Q1: What is the state of the art regarding the application of prescriptive maintenance for analyzing and solving problems related to minimizing machine downtime in the biopharmaceutical industry?

Q2: Are there studies that use prescriptive maintenance in the biopharmaceutical industry with deep learning techniques?

Q3: What are the opportunities and challenges in applying prescriptive maintenance as an analysis tool in activities related to the biopharmaceutical industry?

### 3.3. Evaluation

At this stage, the most relevant documents were selected from all the works found. By focusing on the abstracts of the identified works, the following exclusion criteria (EC) were applied:

- CE1: Works prior to 2018, similar studies, book chapters that do not represent the initial research of this article.
- CE2: Studies that do not involve techniques applicable to the maintenance of equipment in Industry in general.
- CE3: Database articles that were not available for download and articles that were already accepted but not yet published.

**Figure 3:**
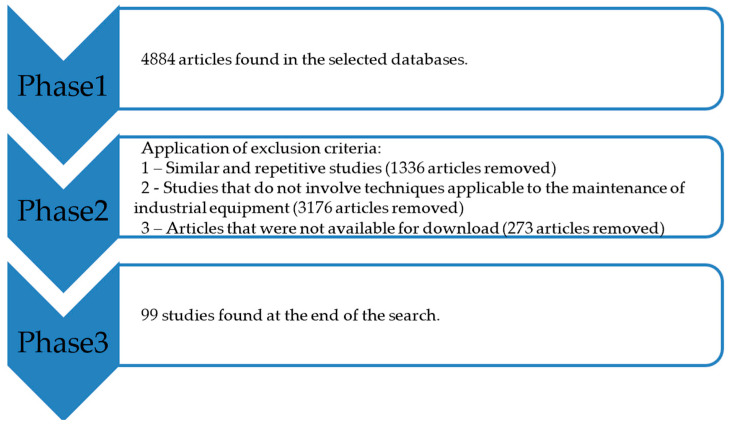
Flowchart of the study selection process.

At the end of this process, 99 of the most relevant studies were selected with the study proposition. Figure 3 illustrates the flow of the methodology followed.

### 3.4. Syntheses

Finally, in the fourth stage, an in-depth reading and analysis of these studies was carried out, seeking to understand the relationships between the results presented and identify patterns, divergences, and research opportunities to answer the three guiding questions proposed. The result obtained from this synthesis will be presented in the next section.

## 4. Results, Discussion, and Perspectives

**Figure 4:**
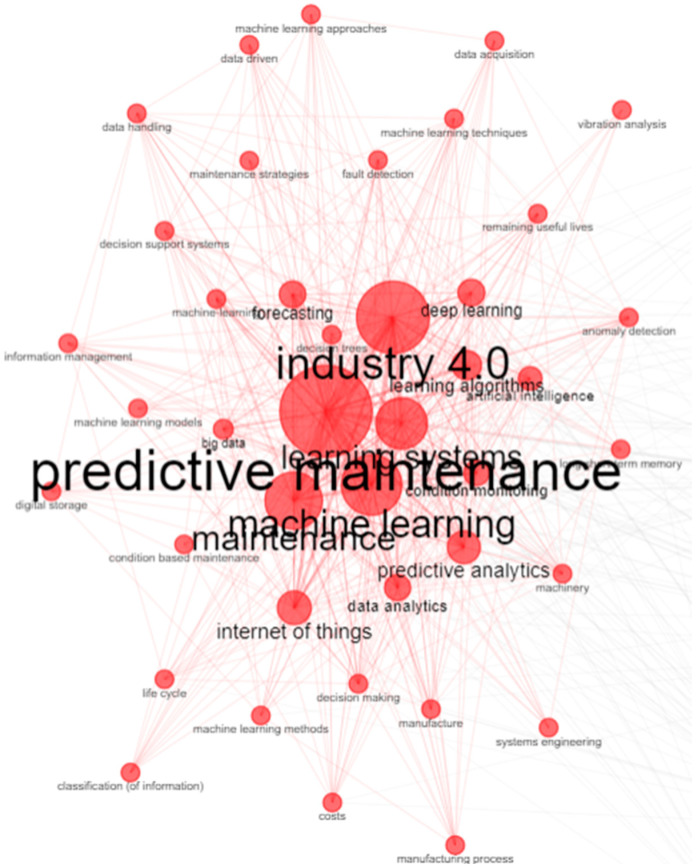
Keyword Cross-Occurrence Network.

After carrying out the research stage, 270 results were found in the strings in Portuguese only on Google Scholar. In English, 197 works were identified in IEEE, 261 works in SCOPUS, 1389 works in ScienceDirect, 263 works in Web of Science, 276 works in the Capes Portal, and 2228 works in Google Scholar. From the cross-occurrence network found, the topic of prescriptive maintenance has been little explored in the literature, despite its great potential in the context of I4.0, according to the graphical analysis of the keyword occurrence network presented in Figure 4.

### 4.1. Results

**Figure 5:**
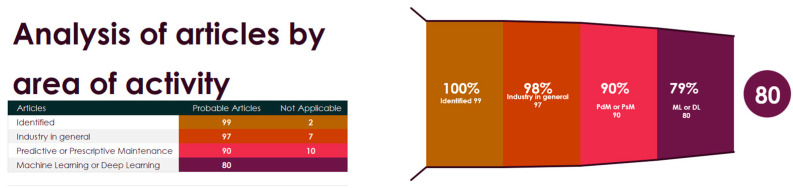
Analysis by area of activity of articles.

The 99 works were read and analyzed in the following categories according to the search: 1—Works applied to industry in general; 2—works that effectively use PdM or PsMtechniques; 3—works that involve ML or DL architectures. In this new filter, they were distributed as follows, according to Figure 5.

**Figure 6:**
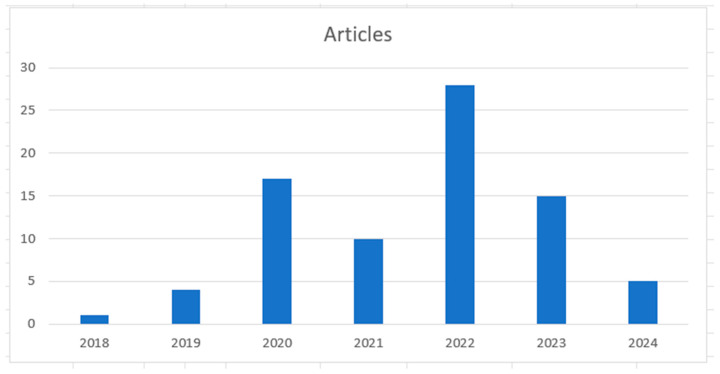
Production of articles per year.

These works were read in their entirety and the relationships, patterns, divergences, and research opportunities identified are presented and analyzed in the subsections below, using the three proposed guiding questions as parameters. Using the area of expertise interface, two articles were excluded because they dealt with strategies or the use of ML techniques without a scope of application in Industry in general. The second filter was applied based on articles that, despite being addressed to the industry in general, did not work on the direct application of PdM and/or PsM in the industry. After this, seven more articles were excluded from the research. The third filter was applied to works that use ML or DL techniques. Of this amount, ten more articles were removed, leaving 80 articles for analysis. By year, it can be seen in Figure 6 that 2022 had the highest production of articles aimed at Industry, using PdM and/or PsM and using ML and/or DL algorithms.

### 4.2. Discussion

Articles [16,17] are aimed at the pharmaceutical industry, taking a PdM approach using ML and DL algorithms, respectively, for different lines of the production process.

Articles [18,19,20,21,22,23,24,25,26] are related to creating a PdM model or reviewing the state of the art in this regard, using several ML algorithms with a main focus on rotating machines, using collected engine vibration data or public data and, for the most part, working with LSTM (long short term memory) network architectures that can solve a problem that affects RNNs (recurrent neural networks), known as gradient disappearance, which occurs with the evolution of learning. The internal architecture of the neural cell presents mechanisms (gates) that control information from the temporal states of the cells, that is, sigmoid and hyperbolic tangent type functions that control the propagation of signals within the cell. The model can select information what will be forgotten or propagated [27].

The next group of articles continues to address PdM analysis of equipment. Articles [28,29,30,31,32,33,34,35,36,37,38,39,40,41] deal with a general review of the topic of predictive maintenance. Articles [42,43,44,45,46,47,48,49,50,51,52,53,54,55,56] demonstrate various applications in different equipment and discuss the type of data that is collected in each type of application. The concept of implementing PdM based on equipment condition is applied [57]. It is important to note that, to implement a predictive model, it is essential that the data be structured. Unstructured data greatly complicate the modeling process. The articles [58,59,60,61,62,63,64,65,66,67,68,69,70,71,72,73,74,75,76,77,78] deal with comparative studies, using different techniques and methodologies with the aim of presenting systems and models to support the decision-making process of PdM. Federated learning (FL) is used, which allows multiple participants to develop a ML model without compromising the privacy and confidentiality of their data [79,80].

Articles [81,82,83,84,85,86,87,88,89,90,91] propose PdM techniques using DL methods. The DL methodology, in this case, is suitable for solving specific tasks. Due to the dynamic nature of industrial processes and environments, transfer learning (TL) is used, taking advantage of knowledge from related tasks and accounting for variations in data distributions, to solve new tasks with little or even no additional labelled data. This approach bypasses the need to retrain a model from scratch for each new configuration and drastically reduces the requirement for labeled data.

**Figure 7:**
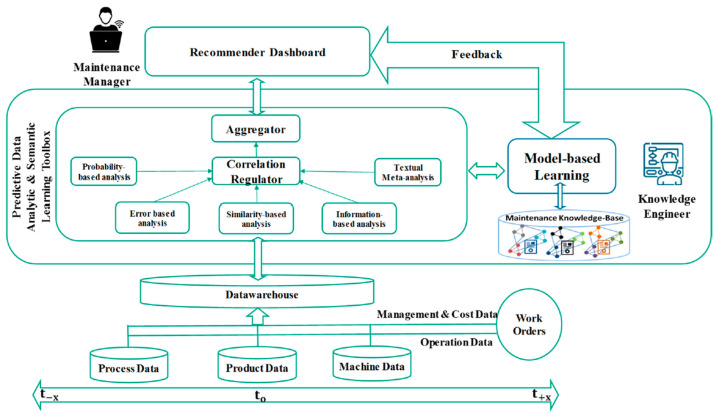
Adapted PRIMA model.

Finally, articles [92,93,94] describe PsM models based on ML and DL architectures for application in the general industry. Article [95] presents a reference model called PriMa-X for implementing PsM. It presents the state of the art of knowledge strategies for the current maintenance base, indicating four steps in the evolution of this knowledge. Present existing maintenance decision support models and existing PsM implementation methods, detailing their gaps. Finally, the article describes the three-layer reference model namely: objectives, changes, and ML and required IT infrastructure. Despite being a reference model only, there is still a gap in the application and presentation of results in industry along the lines of the I4.0 standard. Article [94] deals with a conceptual project that aims to integrate production planning and control (PPC) and maintenance planning and control (MPC). An integrated approach is presented that establishes the link between the production control model and the introduction of prescriptive maintenance model (PriMa). Traditionally, PPC and MPC systems are worked separately from each other. Considering PriMa, various aspects of data-driven maintenance planning and the missing link with autonomous production control are discussed. The authors defend a knowledge-based maintenance (KBM) model. According to them, the presented architecture of PriMa involves three interconnected systems, namely: PsM system, autonomous production control system, and production planning system. Finally, the article only presents a proposed model without any practical application, and a greater definition of how to apply PsM and its operation. Figure 7 shows the architecture of the PRIMA model.

### 4.3. Perspectives

Most of the applications discussed previously are for use in industry in general, with a few articles focused exclusively on the pharmaceutical industry. This does not invalidate the application of all the techniques and models explained in the articles, considering their peculiarities. The use of LSTM algorithms has been widely applied in PdM solutions in rotating machines. The origin and nature of the data (structured and unstructured) has been the focus of discussions in another group of articles that will facilitate the choice of the specific technique or algorithm for each application. Another topic discussed could be the emphasis on systems and models to support the decision-making process, using ML techniques, especially FL, to solve specific tasks within the industry, DL techniques have also been used, especially TL. Finally, there is a group of articles, which propose PsM models based on ML and DL architectures for applications in the industry in general.

**Figure 8:**
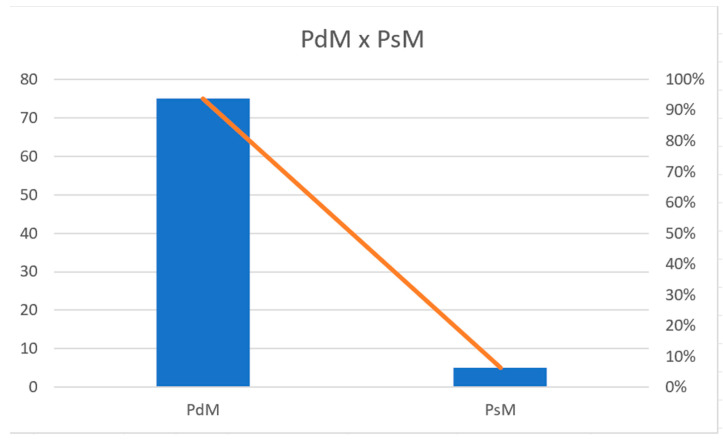
Percentage of Predictive × Prescriptive articles.

Based on the presentation of the articles, we turn to the guiding questions of the article, namely: Q1: What is the state of the art in relation to the application of PsM for the analysis and solution of problems related to minimizing machine downtime in the biopharmaceutical industry? It is true to say that PsM solutions accounted for 6.25% in relation to PdM solutions (93.75%) as shown in Figure 8. There is a need for greater investment in the use of appropriate techniques and models involving PsM, as well as defining and integrating factory floor data from IoT sensors into production assets, with process data from production equipment from programmable logic controllers (PLC) and their human-machine interfaces (HMI) added to the data of production planning and maintenance control to generate a prescriptive system to aid production decision-making.

Q2: Are there studies that use PsM in the biopharmaceutical Industry with DL techniques? No specific studies of PsM techniques applied in the biopharmaceutical Industry were found. Q3: What are the opportunities and challenges in applying PsM as an analysis tool in activities related to the biopharmaceutical industry? The aims of applying PsM in the biopharmaceutical industry in view of the current I4.0 tools are numerous, mainly aiming to integrate systems, have better treatment of factory floor data, and optimize production in a pharmaceutical plant in compliance with the requirements of the regulatory bodies of this industry. The new needs of I5.0 must now be considered, especially in terms of data security and industry sustainability, among other factors.

## 5. Conclusions

This work aimed to evaluate current knowledge about the challenges and opportunities of the biopharmaceutical industry in the application of PsM in the context of I4.0. The results showed that there are some proposed models, but no concrete applications aimed at equipment in the biopharmaceutical industry. There is too much emphasis on the urgency, importance, and application of this topic in the biopharmaceutical industry. Noteworthy challenges include a need for investment by companies in new technologies with the aim of obtaining the best productivity and economics of production, as well as working to change the culture of the biopharmaceutical industry, as there is still a lot of resistance both from people who work in production and in maintenance, and production planning and maintenance of the advances obtained through the use of these new technologies, mainly in terms of safety, assertiveness, economy, and better productivity for these industry actors.

With the growth of factories and the need to provide greater storage, better processing of production data, and maintenance of the organization, there is scope for action and investment to be made in AI with neural networks and DL techniques.

There are many articles that validate the need to consolidate the concepts of PdM aligned with ML techniques with practical applications in the biopharmaceutical industry, which will be a fundamental step and foundation for the implementation of PsM, which has data processing in its structure. Production, factory floor, and corporate data can guide the planner’s decision-making in terms of the prediction of failures occurring in the plant.

The perspectives show several applications aimed at the industry in general using PdM and PsM models with some techniques providing better results in specific systems. They point to the use and integration of data processing techniques from various sources in the production process, with the aim of advancing the real implementation of PsM. Implicit in the articles presented is the need to integrate current technologies with PPC and MPC systems, facilitating decision-making and optimizing the production process.

A limitation of this work is that many articles from our bibliographic research were not accessible. The Google Scholar database does not have filters that facilitate a more precise search for keywords. Additionally, some articles lack specification about the origin of the data used, as well as clarity of the tools adopted and detailed specification of data from test equipment used in research.

As future research, it is suggested to invest in the development of PsM models, using the current tools available, applying the new demands of I5.0 to the assets of the biopharmaceutical industry, with a greater focus on providing more security to data networks and sustainability, seeking to meet sustainable development goals (SDG) when dealing with Industry assets. Finally, seeking to implement a symbiotic/collaborative relationship between the human being and the machine, considering issues such as defining data entry and analysis methods, and the integration of technologies and industry players would be valuable.

## Data Availability

No new data were created or analyzed in this study.

## References

[1] Silva F., Amorim M., Resende D. (2020). Um estudo sobre a aplicação dos conceitos e elementos da indústria 4.0 na produção de biomedicamentos. Rev. Prod. Online.

[2] Albrecht I., Rhoden S.A., Pamphile J.A. (2015). Indústria Biofarmacêutica E Seu Processo Produtivo. Evidência.

[3] Silva F., Resende D., Amorim M., Borges M. (2020). A Field Study on the Impacts of Implementing Concepts and Elements of Industry 4.0 in the Biopharmaceutical Sector. J. Open Innov. Technol. Mark. Complex..

[4] Lima A.L.d.C.D., Aranha V.M., Carvalho C.J.d.L., Nascimento E.G.S. (2021). Smart Predictive Maintenance for High-Performance Computing Systems: A Literature Review. J. Supercomput..

[5] Macedo L.C. (2020). Manutenção Preditiva no Contexto da Indústria 4.0: Um Modelo Preditivo em Uma Fábrica do Ramo Metalúrgico. Bachelor’s Thesis.

[6] Creswell J.W. (2010). Projeto de Pesquisa: Metodos Qualitativo, Quantitativo e Misto.

[7] Schwab K. (2016). A Quarta Revolucao Industrial.

[8] Özdemir V., Hekim N. (2018). Birth of Industry 5.0: Making Sense of Big Data with Artificial Intelligence, “the Internet of Things” and Next-Generation Technology Policy. Omics J. Integr. Biol..

[9] Zhang C., Wang Z., Zhou G., Chang F., Ma D., Jing Y., Cheng W., Ding K., Zhao D. (2023). Towards New-Generation Human-centric Smart Manufacturing in Industry 5.0: A Systematic Review. Adv. Eng. Inform..

[10] Coelho P., Bessa C., Landeck J., Silva C. (2022). Industry 5.0: The Arising of a Concept. Procedia Comput. Sci..

[11] Nahavandi S. (2019). Industry 5.0—A Human-Centric Solution. Sustainability.

[12] Maddikunta P.K.R., Pham Q.V., Prabadevi B., Deepa N., Dev K., Gadekallu T.R., Ruby R., Liyanage M. (2022). Industry 5.0: A Survey on Enabling Technologies and Potential Applications. J. Ind. Inf. Integr..

[13] Viana H.R.G. (2008). PCM, Planejamento e Controle de Manutenção.

[14] Santos E. (2019). Manutenção Aeronáutica Preditiva—Procedimentos, Técnicas E Business Models. Master’s Thesis.

[15] Faceli K., Lorena A.C., Gama J., Almeida T.A.D., Carvalho A.C.P.L.F. (2021). Inteligência Artificial—Uma Abordagem de Aprendizado de Máquina.

[16] Azab E., Nafea M., Shihata L.A., Mashaly M. (2021). A Machine-Learning-Assisted Simulation Approach for Incorporating Predictive Maintenance in Dynamic Flow-Shop Scheduling. Appl. Sci..

[17] Kavasidis I., Lallas E., Gerogiannis V.C., Charitou T., Karageorgos A. (2023). Predictive Maintenance in Pharmaceutical Manufacturing Lines Using Deep Transformers. Procedia Comput. Sci..

[18] Zaro E.M., Webber C.G. (2022). Estudo de Caso de Desenvolvimento de Sistema Para Manutenção Preditiva 4.0. Rev. Prod. Online.

[19] Achouch M., Dimitrova M., Dhouib R., Ibrahim H., Adda M., Sattarpanah Karganroudi S., Ziane K., Aminzadeh A. (2023). Predictive Maintenance and Fault Monitoring Enabled by Machine Learning: Experimental Analysis of a TA-48 Multistage Centrifugal Plant Compressor. Appl. Sci..

[20] Magadán L., Suárez F.J., Granda J.C., delaCalle F.J., García D.F. (2023). A Robust Health Prognostics Technique for Failure Diagnosis and the Remaining Useful Lifetime Predictions of Bearings in Electric Motors. Appl. Sci..

[21] Vallim Filho A.R.d.A., Farina Moraes D., Bhering de Aguiar Vallim M.V., da Silva L.S., da Silva L.A. (2022). A Machine Learning Modeling Framework for Predictive Maintenance Based on Equipment Load Cycle: An Application in a Real World Case. Energies.

[22] Mohan R., Roselyn J.P., Uthra R.A. (2023). LSTM Based Artificial Intelligence Predictive Maintenance Technique for Availability Rate and OEE Improvement in a TPM Implementing Plant through Industry 4.0 Transformation. J. Qual. Maint. Eng..

[23] Cardoso D., Ferreira L. (2021). Application of Predictive Maintenance Concepts Using Artificial Intelligence Tools. Appl. Sci..

[24] Dalzochio J., Kunst R., Pignaton E., Binotto A., Sanyal S., Favilla J., Barbosa J. (2020). Machine Learning and Reasoning for Predictive Maintenance in Industry 4.0: Current Status and Challenges. Comput. Ind..

[25] Zenisek J., Holzinger F., Affenzeller M. (2019). Machine Learning Based Concept Drift Detection for Predictive Maintenance. Comput. Ind. Eng..

[26] Shrivastava M., Singhal P., Bhuvana J. (2023). Integrating Sensor Data and Machine Learning for Predictive Maintenance in Industry 4.0. Proc. Eng. Sci..

[27] Olah C. Understanding LSTM Networks. https://colah.github.io/posts/2015-08-Understanding-LSTMs.

[28] Leukel J., González J., Riekert M. (2021). Adoption of Machine Learning Technology for Failure Prediction in Industrial Maintenance: A Systematic Review. J. Manuf. Syst..

[29] Grubisic V.V.F., Aguiar J.P.F., Simeu-Abazi Z. A Review on Intelligent Predictive Maintenance: Bibliometric Analysis and New Research Directions. Proceedings of the 2020 International Conference on Control, Automation and Diagnosis (ICCAD).

[30] Namuduri S., Narayanan B.N., Davuluru V.S.P., Burton L., Bhansali S. (2020). Review—Deep Learning Methods for Sensor Based Predictive Maintenance and Future Perspectives for Electrochemical Sensors. J. Electrochem. Soc..

[31] Carvalho T.P., Soares F.A.A.M.N., Vita R., Francisco R.d.P., Basto J.P., Alcalá S.G.S. (2019). A Systematic Literature Review of Machine Learning Methods Applied to Predictive Maintenance. Comput. Ind. Eng..

[32] Jha U.C., Sai R.J., Reddy M., Singh A. (2022). Analysis of Predictive Maintenance in Industry 4.0: A review. Int. J. Mech. Eng..

[33] Kiran M.B. Smart Preventive Maintenance—A Review. Proceedings of the 12th Annual International Conference on Industrial Engineering and Operations Management.

[34] Aquino E.L.R.D., Mollo Neto M., Bernardo C.H.C., Morais F.J.d.O., Santos P.S.B.d. (2020). Ferramentas de Manutenção Preditiva de Motores Diesel: Uma Revisão Bibliográfica Sistemática. Res. Soc. Dev..

[35] Maktoubian J., Taskhiri M.S., Turner P. (2021). Intelligent Predictive Maintenance (Ipdm) in Forestry: A Review of Challenges and Opportunities. Forests.

[36] Keleko A.T., Kamsu-Foguem B., Ngouna R.H., Tongne A. (2022). Artificial Intelligence and Real-Time Predictive Maintenance in Industry 4.0: A Bibliometric Analysis. AI Ethics.

[37] Zonta T., da Costa C.A., da Rosa Righi R., de Lima M.J., da Trindade E.S., Li G.P. (2020). Predictive Maintenance in the Industry 4.0: A systematic Literature Review. Comput. Ind. Eng..

[38] Cheng X., Chaw J.K., Goh K.M., Ting T.T., Sahrani S., Ahmad M.N., Abdul Kadir R., Ang M.C. (2022). Systematic Literature Review on Visual Analytics of Predictive Maintenance in the Manufacturing Industry. Sensors.

[39] Çınar Z.M., Nuhu A.A., Zeeshan Q., Korhan O. (2020). Machine Learning in Predictive Maintenance towardsSustainable Smart Manufacturing in Industry 4.0. Sustainability.

[40] Surucu O., Gadsden S.A., Yawney J. (2023). Condition Monitoring Using Machine Learning: A Review of Theory, Applications, and Recent Advances. Expert Syst. Appl..

[41] Vaidyanathan B. A Review of Acoustic & Vibration Based Real-Time Monitoring Predictive Maintenance. Proceedings of the 1st Advanced Manufacturing Student Conference (AMSC21).

[42] Kane A., Kore A., Khandale A., Nigade S., Joshi P.P. (2019). Predictive Maintenance Using Machine Learning. Comput. Eng. Dep. Pune Inst. Comput. Technol..

[43] Hoffmann M.W., Wildermuth S., Gitzel R., Boyaci A., Gebhardt J., Kaul H., Amihai I., Forg B., Suriyah M., Leibfried T. (2020). Integration of Novel Sensors and Machine Learning for Predictive Maintenance in Medium Voltage Switchgear to Enable the Energy and Mobility Revolutions. Sensors.

[44] Deshmukh M., Dumbre R., Anekar S., Kulkarni H., Pawar S. (2021). Condition Monitoring and Predictive Maintenance of Process Equipments. ITM Web Conf..

[45] Abidi M.H., Umer U., Mohammed M.K., Aboudaif M.K., Alkhalefah H. (2020). Automated Maintenance Data Classification Using Recurrent Neural Network: Enhancement by Spotted Hyena-Based Whale Optimization. Mathematics.

[46] Consilvio A., Solís-Hernández J., Jiménez-Redondo N., Sanetti P., Papa F., Mingolarra-Garaizar I. (2020). On Applying Machine Learning and Simulative Approaches to Railway Asset Management: The Earthworks and Track Circuits Case Studies. Sustainability.

[47] De Simone L., Caputo E., Cinque M., Galli A., Moscato V., Russo S., Cesaro G., Criscuolo V., Giannini G. (2023). LSTM-Based Failure Prediction for Railway Rolling Stock Equipment. Expert Syst. Appl..

[48] Küfner T., Döpper F., Müller D., Trenz A. (2021). Predictive Maintenance: Using Recurrent Neural Networks for Wear Prognosis in Current Signatures of Production Plants. Int. J. Mech. Eng. Robot. Res..

[49] Phan T.L.J., Gehrhardt I., Heik D., Bahrpeyma F., Reichelt D. (2022). A Systematic Mapping Study on Machine Learning Techniques Applied for Condition Monitoring and Predictive Maintenance in the Manufacturing Sector. Logistics.

[50] Velasquez D., Perez E., Oregui X., Artetxe A., Manteca J., Mansilla J.E., Toro M., Maiza M., Sierra B. (2022). A Hybrid Machine-Learning Ensemble for Anomaly Detection in Real-Time Industry 4.0 Systems. IEEE Access.

[51] Almobarek M., Mendibil K., Alrashdan A. (2023). Predictive Maintenance 4.0 for Chilled Water System at Commercial Buildings: A Methodological Framework. Buildings.

[52] Nikfar M., Bitencourt J., Mykoniatis K. (2022). A Two-Phase Machine Learning Approach for Predictive Maintenance of Low Voltage Industrial Motors. Procedia Comput. Sci..

[53] Silva A.A., Henrique Corrêa de Souza P., Xavier de Freitas R., Orientador P., Ribeiro Silva S. (2022). Predição De Falhas Mecânicas De Motores Elétricos Industriais Através De Análise Sinais Mecânicos Com Sistema De Inteligência Artificial. https://repositorio.animaeducacao.com.br/items/4e9153fa-c332-4edb-9e6d-53b5cfa92f9f.

[54] Lemos T.D., De Souza L.A.Z. (2022). Abordagem Preditiva de Quebras Baseada Em Logs de Eventos Na Indústria Automotiva. Rev. Eng. Pesqui. Apl..

[55] Manchadi O., Ben-Bouazza F.E., Jioudi B. (2023). Predictive Maintenance in Healthcare System: A Survey. IEEE Access.

[56] Drakaki M., Karnavas Y.L., Tziafettas I.A., Linardos V., Tzionas P. (2022). Machine Learning and Deep Learning Based Methods Toward Industry 4.0 Predictive Maintenance in Induction Motors: A State of the Art Survey. J. Ind. Eng. Manag..

[57] Manjare A.A., Patil B.G. A Review: Condition Based Techniques and Predictive Maintenance for Motor. Proceedings of the 2021 International Conference on Artificial Intelligence and Smart Systems (ICAIS).

[58] Karuppusamy P. (2021). Machine Learning Approach to Predictive Maintenance in Manufacturing Industry—A Comparative Study. J. Soft Comput. Paradig..

[59] Mattioli J., Perico P., Robic P.O. Artificial Intelligence Based Asset Management. Proceedings of the 2020 IEEE 15th International Conference of System of Systems Engineering (SoSE).

[60] Achouch M., Dimitrova M., Ziane K., Sattarpanah Karganroudi S., Dhouib R., Ibrahim H., Adda M. (2022). On Predictive Maintenance in Industry 4.0: Overview, Models, and Challenges. Appl. Sci..

[61] Coandǎ P., Avram M., Constantin V. (2020). A State of the Art of Predictive Maintenance Techniques. IOP Conf. Ser. Mater. Sci. Eng..

[62] Arena S., Florian E., Zennaro I., Orrù P.F., Sgarbossa F. (2022). A Novel Decision Support System for Managing Predictive Maintenance Strategies Based on Machine Learning Approaches. Saf. Sci..

[63] Bilal Yıldız G., Soylu B. (2023). Integrating Preventive and Predictive Maintenance Policies with System Dynamics: A Decision Table Approach. Adv. Eng. Inform..

[64] Mohammed B., Nadia M., Mourad Z. (2022). Multi-Criteria Analysis of Diagnostic and Prognostic Models for Predictive Maintenance. E3S Web Conf..

[65] Calabrese M., Cimmino M., Fiume F., Manfrin M., Romeo L., Ceccacci S., Paolanti M., Toscano G., Ciandrini G., Carrotta A. (2020). SOPHIA: An Event-Based IoT and Machine Learning Architecture for Predictive Maintenance in Industry 4.0. Information.

[66] Rahman A., Pasanbu E., Nugraha Y., Khair F., Soebandrija K.E.N., Wijaya D.I. (2020). Industry 4.0 and Society 5.0 through Lens of Condition Based Maintenance (CBM) and Machine Learning of Artificial Intelligence (MLAI). IOP Conf. Ser. Mater. Sci. Eng..

[67] Gayialis S.P., Kechagias E.P., Konstantakopoulos G.D., Papadopoulos G.A. (2022). A Predictive Maintenance System for Reverse Supply Chain Operations. Logistics.

[68] Franco I.T., de Figueiredo R.M. (2023). Predictive Maintenance: An Embedded System Approach. J. Control Autom. Electr. Syst..

[69] Abidi M.H., Mohammed M.K., Alkhalefah H. (2022). Predictive Maintenance Planning for Industry 4.0 Using Machine Learning for Sustainable Manufacturing. Sustainability.

[70] Kiangala K.S., Wang Z. (2020). An Effective Predictive Maintenance Framework for Conveyor Motors Using Dual Time-Series Imaging and Convolutional Neural Network in an Industry 4.0 Environment. IEEE Access.

[71] Tortora A.M.R., Veneroso C.R., Di Pasquale V., Riemma S., Iannone R. (2024). Machine Learning for Failure Prediction: A Cost-Oriented Model Selection. Procedia Comput. Sci..

[72] Justus V., Kanagachidambaresan G.R. (2024). Machine Learning Based Fault-Oriented Predictive Maintenance in Industry 4.0. Int. J. Syst. Assur. Eng. Manag..

[73] Mallidis I., Yakavenka V., Konstantinidis A., Sariannidis N. (2021). A Goal Programming-Based Methodology for Machine Learning Model Selection Decisions: A Predictive Maintenance Application. Mathematics.

[74] Nacchia M., Fruggiero F., Lambiase A., Bruton K. (2021). A Systematic Mapping of the Advancing Use of Machine Learning Techniques for Predictive Maintenance in the Manufacturing Sector. Appl. Sci..

[75] Wahid A., Breslin J.G., Intizar M.A. (2022). Prediction of Machine Failure in Industry 4.0: A Hybrid CNN-LSTM Framework. Appl. Sci..

[76] Gorski E.G., Loures E.d.F.R., Santos E.A.P., Kondo R.E., Martins G.R.D.N. (2022). Towards a Smart Workflow in CMMS/EAM Systems: An Approach Based on ML and MCDM. J. Ind. Inf. Integr..

[77] Züfle M., Moog F., Lesch V., Krupitzer C., Kounev S. (2022). A Machine Learning-Based Workflow for Automatic Detection of Anomalies in Machine Tools. ISA Trans..

[78] Yeardley A.S., Ejeh J.O., Allen L., Brown S.F., Cordiner J. (2022). Integrating Machine Learning Techniques into Optimal Maintenance Scheduling. Comput. Chem. Eng..

[79] Bemani A., Björsell N. (2022). Aggregation Strategy on Federated Machine Learning Algorithm for Collaborative Predictive Maintenance. Sensors.

[80] Pruckovskaja V., Weissenfeld A., Heistracher C., Graser A., Kafka J., Leputsch P., Schall D., Kemnitz J. Federated Learning for Predictive Maintenance and Quality Inspection in Industrial Applications. Proceedings of the 2023 Prognostics and Health Management Conference (PHM).

[81] Sperandio Nascimento E.G., Liang J.S., Figueiredo I.S., Guarieiro L.L.N. (2022). T4pdm: A Deep Neural Network Based on the Transformer Architecture for Fault Diagnosis of Rotating Machinery. SSRN Electron. J..

[82] Murugiah P., Muthuramalingam A., Anandamurugan S. (2023). A Design of Predictive Manufacturing System in IoT-Assisted Industry 4.0 Using Heuristic-Derived Deep Learning. Int. J. Commun. Syst..

[83] Yan P., Abdulkadir A., Luley P.P., Rosenthal M., Schatte G.A., Grewe B.F., Stadelmann T. (2024). A Comprehensive Survey of Deep Transfer Learning for Anomaly Detection in Industrial Time Series: Methods, Applications, and Directions. IEEE Access.

[84] Serradilla O., Zugasti E., Rodriguez J., Zurutuza U. (2022). Deep Learning Models for Predictive Maintenance: A Survey, Comparison, Challenges and Prospects. Appl. Intell..

[85] Maher Y., Danouj B. (2020). Survey on Deep Learning Applied to Predictive Maintenance. Int. J. Electr. Comput. Eng..

[86] Hassan M.U., Steinnes O.M.H., Gustafsson E.G., Løken S., Hameed I.A. (2023). Predictive Maintenance of Norwegian Road Network Using Deep Learning Models. Sensors.

[87] Azari M.S., Flammini F., Santini S., Caporuscio M. (2023). A Systematic Literature Review on Transfer Learning for Predictive Maintenance in Industry 4.0. IEEE Access.

[88] Ruan H., Dorneanu B., Arellano-Garcia H., Xiao P., Zhang L. (2022). Deep Learning-Based Fault Prediction in Wireless Sensor Network Embedded Cyber-Physical Systems for Industrial Processes. IEEE Access.

[89] Jamwal A., Agrawal R., Sharma M. (2022). Deep Learning for Manufacturing Sustainability: Models, Applications in Industry 4.0 and Implications. Int. J. Inf. Manag. Data Insights.

[90] Jaenal A., Ruiz-Sarmiento J.R., Gonzalez-Jimenez J. (2024). MachNet, a General Deep Learning Architecture for Predictive Maintenance within the Industry 4.0 Paradigm. Eng. Appl. Artif. Intell..

[91] Wang L., Zhu Z., Zhao X. (2024). Dynamic Predictive Maintenance Strategy for System Remaining Useful Life Prediction via Deep Learning Ensemble Method. Reliab. Eng. Syst. Saf..

[92] Santiago R.A.d.F., Barbosa N.B., Mergulhão H.G., Carvalho T.F.d., Santos A.A.B., Medrado R.C., Filho J.B.d.M., Pinheiro O.R., Nascimento E.G.S. (2024). Data-Driven Models Applied to Predictive and Prescriptive Maintenance of Wind Turbine: A Systematic Review of Approaches Based on Failure Detection, Diagnosis, and Prognosis. Energies.

[93] Goby N., Brandt T., Neumann D. (2023). Deep Reinforcement Learning with Combinatorial Actions Spaces: An Application to Prescriptive Maintenance. Comput. Ind. Eng..

[94] Glawar R., Ansari F., Kardos C., Matyas K., Sihn W. (2019). Conceptual Design of an Integrated Autonomous Production Control Model in Association with a Prescriptive Maintenance Model (Prima). Procedia CIRP.

[95] Nemeth T., Ansari F., Sihn W., Haslhofer B., Schindler A. (2018). PriMa-X: A Reference Model for Realizing Prescriptive Maintenance and Assessing Its Maturity Enhanced by Machine Learning. Procedia CIRP.

